# The association of abnormal chest X-ray findings with mortality in patients requiring mechanical ventilation in mulago hospital general intensive care unit

**DOI:** 10.1186/2197-425X-3-S1-A674

**Published:** 2015-10-01

**Authors:** HDG Ssemmanda, J Ejoku, TS Luggya, C Lubulwa

**Affiliations:** Makerere University College of Health Sciences, Anaesthesia and Critical Care, Kampala, Uganda

## Background

Chest X-rays are a relatively cheap investigative tool that aids in early detection of cardiopulmonary abnormalities and this translates in better mortality outcomes and reduced length of stay (Henschke, Yankelevitz et al. 1996). According to a study done in Mulago general ICU by Ssemogerere et al, more than half of admitted patients needed a form of respiratory support especially mechanical ventilation. The same study also quoted a high mortality rate in these patients. (Ssemogerere. L 2014)

## Objective

To determine the magnitude of abnormal chest x-rays in patients requiring mechanical ventilation and the association of these abnormalities with mortality

## Methodology

Upon approval from school of medicine research and ethics committee, we conducted a cross-sectional study in the general ICU of Mulago. A total of 91 patients were sequentially recruited into the study. Data was retrieved on admission and on discharge. Data entry was done using EpiData 3.1Range. Analysis was done using STATA.

## Results

The majority of patients admitted for mechanical ventilation were from trauma center at 30.77%. Consequently CNS impairment with concomitant respiratory disease was the biggest indication for mechanical ventilation at 29.67%. Notably 73.56% of study CXRs were abnormal and lung parenchyma abnormalities were the biggest abnormality seen by the radiologists. Among these, bronchopneumonia contributed 23%. Of the 87 patients analyzed 41died and of these, 34 patients had abnormal CXRs. Patients with MEWS score ≥5 had an incidence risk ratio of 3.29.

## Conclusions and Recommendations

Most of the patients admitted for mechanical ventilation had an abnormality in the lungs and these abnormalities positively correlate with mortality. Due to the positive correlation between patients with high MEWS, this score can be used to predict mortality in this patient population. Patients who present to the ICU for mechanical ventilation should have a chest X-ray done within 24 hours of initiating the mechanical ventilation. A portable x-ray machine should be deployed in the ICU of Mulago hospital and a radiographer be put on ICU call roster.
